# Damage Evolution Mechanism of Railway Wagon Bogie Adapter 1035 Steel and Damage Parameter Calibration Based on Gursone–Tvergaarde–Needleman Model

**DOI:** 10.3390/ma17205070

**Published:** 2024-10-17

**Authors:** Jiachuan Yin, Xiaomin Huang, Guangzhi Ma, Changzhe Song, Xuefeng Tang, Hongchao Ji

**Affiliations:** 1 College of Mechanical Engineering, North China University of Science and Technology, Tangshan 063210, China; 15932560708@163.com (J.Y.); hxm8606@126.com (X.H.); 2 Yanching Institute of Technology, Langfang 065201, China; 3 China MCC22 Group Corporation Ltd., Tangshan 063035, China; mcc22song@126.com; 4 State Key Laboratory of Materials Processing and Die & Mould Technology, Huazhong University of Science and Technology, 1037 Luoyu Road, Wuhan 430074, China; xftang@hust.edu.cn

**Keywords:** AISI 1035 steel, GTN model, damage evolution analysis, void volume fraction, ductile fracture

## Abstract

As a critical component of a train, the railway wagon bogie adapter has higher quality requirements. During the forging process, external loads can induce voids, cracks, and other defects in the forging, thereby reducing its service life. Hence, studying the damage behavior of the forging material, specifically AISI 1035 steel, becomes crucial. This study involved obtaining stress–strain curves for AISI 1035 steel through uniaxial tensile tests at temperatures of 900 °C, 1000 °C, and 1100 °C, with strain rates of 0.1 s^−1^, 1 s^−1^, and 10 s^−1^. Subsequently, SEM was used to observe samples at various deformation stages. The damage parameters, $q_{1}$, $\;q_{2}$ and $q_{3}$ in the GTN model “a computational model used to analyze and simulate material damage which can effectively capture the damage behavior of materials under different loading conditions” were then calibrated using the Ramberg–Osgood model and stress–strain curve fitting. Image Pro Plus software v11.1 quantified the sample porosity as $f_{0}$, $f_{n}$, $f_{c}$ and $f_{F}$. A finite element model was established to simulate the tensile behavior of the AISI 1035 steel samples. By comparing the damage parameters of $f_{0}$, $f_{n}$, $f_{c}$ and $f_{F}$ obtained by the finite element method and experimental method, the validity of the damage parameters obtained by the finite element inverse method could be verified.

## 1. Introduction

The railway wagon bogie adapter is usually connected to the car body and the chassis, which is used to disperse and transfer the weight of the train car so as to reduce the local pressure on the track. This ensures the train is evenly distributed on the track and its stability during operation, avoids the car shaking or falling off, and extends the service life of the track through a solid connection. In the aerospace, automotive and medical device fields, as well as in other demanding manufacturing areas, especially those requiring custom and complex parts, single-point incremental forming (SPIF) will play an increasingly important role in the future of manufacturing as a flexible and efficient manufacturing technology. Single-point incremental forming belongs to the category of additive manufacturing and plastic forming. The basic principle is to gradually apply force to the material through a controlled tool (usually a conical or spherical tool), which causes plastic deformation of the material at each point of application, and finally forms the desired three-dimensional shape. Many scholars have also carried out much of research on this technology.

Liu et al. [1] have assessed the sustainability of advancements in progressive sheet metal forming technology. Their research indicated that when evaluating mold costs and mass production within the single-point progressive forming process, significant advantages and promising prospects emerge for batch sizes under 1000. Kumar et al. [2] investigated the formability of dissimilar aluminum alloy sheets AA5083 and AA7075 welded using friction stirring in single-point incremental forming. Using ultimate tensile strength (UTS) and elongation (PE) as key metrics, their findings revealed that a combination of lower rotation speed, increased welding speed, and an appropriately sized tool head enhances the forming accuracy of the workpiece. Wu et al. [3] introduced a novel parametric multi-step machining path, demonstrating that adjusting various influencing factors can achieve higher geometric accuracy within ±0.6 mm.

As an important force part of the bogie, the quality of the railway wagon bogie adapter directly determines the safety, reliability and stability of the train operation. However, during the process of high temperature forming, the external load will nucleate microvoids in the vicinity of grain boundaries, form second-phase particles and inclusions, and generate new voids [4]. The accumulation of these voids can ultimately result in fracture failure of the forging. Therefore, understanding the evolution mechanism of meso-damage in AISI 1035 steel during thermal deformation holds significant importance in enhancing the quality and performance of forgings.

The macroscopic properties of the material are determined by its microstructure, and the microstructure characteristics of the steel will also change correspondingly under high temperature and external load. Therefore, neither macroscopic damage mechanics nor microscopic damage mechanics [5,6] can be applied to properly analyze its mechanical behavior.

The mesoscopic damage mechanics approach takes into account the advantages of the above two methods. Mesoscopic damage mechanics can be divided into two main mechanisms: for brittle materials, it is mainly micro-crack damage [7]; for ductile materials, it is microvoid damage. In the realm of mesoscopic damage mechanics, the GTN model is widely adopted in academic research. From a microscopic standpoint, the damage process involves the nucleation, growth, and aggregation of voids [8].

The Gursone–Tvergaarde–Needleman (GTN) [9] model of ductile damage has a unique advantage in the research of material forming processes. Numerous scholars have conducted research on the void damage model. Gurson [10] first proposed two finite size cell models, cylindrical and three-dimensional. However, the Gurson model is only suitable for ideal isotropic elastoplastic materials. Then, Needleman and Tvergaard [11,12,13] improved the Gurson model, forming a classical porous plastic damage model, called the Gursone–Tvergaarde–Needleman (GTN) model. In this model, parameters are introduced into the constitutive equation, and the influences of stress, strain and void interaction in the plastic damage process are fully considered. The GTN model has a good prediction effect on metal ductile damage [14,15,16,17], providing a new way for studying the toughness of material damage.

Many scholars have predicted the material damage behavior well through the GTN model. Yin et al. [18] conducted a tensile test on a notched round bar sample of Q355D steel and utilized the calibrated GTN model to simulate the tensile behavior of the steel plate. They found that the results agree well and that the fracture of the steel plate was predicted accurately. Yoshida et al. [19] set GTN model parameters for 316L stainless steel cold-working materials and simulated the fracture behavior of plate samples. The results showed that toughness fracture assessment and the GTN model were suitable for BWR reactors affected by neutron irradiation. In Wu’s study [20], negative stress triaxiality and fracture effects were incorporated into a modified GTN model. This modified model was subsequently implemented in finite element software to simulate spinning processes on 2024-T351 aluminum alloy. By comparing simulation results with experimental data, an error of only 8.36% in maximum thinning rates was observed, validating the enhanced GTN model’s suitability for spinning applications. Yan et al. [21] used the GTN damage model to study the ductile failure of S700 material under cold forming, and calibrated the parameters q1, q2 and q3 in the yield surface of the GTN model. Brahim [22] proposed an optimization method that utilizes the maximum stress at various notch depths to predict fracture initiation. The GTN model was employed to simulate tensile fractures in samples with varying notch depths, confirming the model’s accuracy. Yildiz [23] effectively utilized the GTN model to forecast the ductile fracture of 6061 aluminum alloy during the forming process. Quan Sun et al. [24] introduced a novel approach for determining the shear-modified GTN parameters proposed by Nahshon and Hutchinson, integrating neural network algorithms with small punch tests. Li et al. [25] employed the GTN model to investigate the evolution of damage in AA7075-T6 aluminum alloy during hot forming. Using the GTN model, Gao et al. [26] investigated the mechanical behavior and material loss during the hot small punch test (T-SPT), and predicted both damage evolution and distribution in automobile B-pillars during hot forming.

With the research and modification of damage models, the application range of GTN model continues to expand. However, the application of the GTN model to AISI 1035 steel has been limited. Therefore, the GTN mesodamage model is introduced into the tensile simulation of AISI 1035 steel to explore the application of the GTN model on the saddle-bearing hot-forging material AISI 1035 steel.

## 2. Experimental Methods

### 2.1. Materials

**Figure 1:**
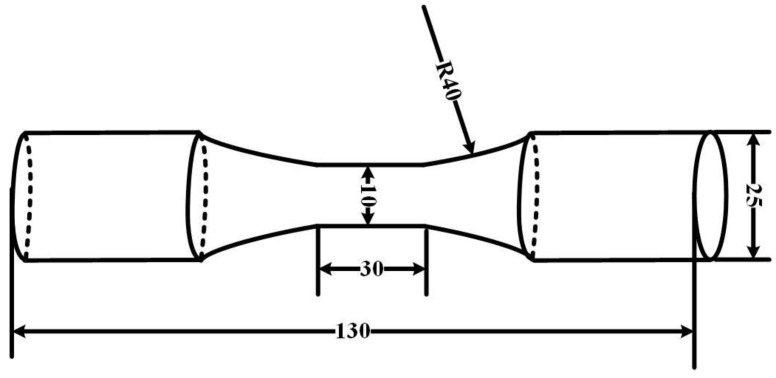
Geometry and size of tensile sample.

The experimental material is AISI 1035 steel. The main components are shown in Table 1, and the dimensions of the tensile sample are illustrated in Figure 1. The total length is 130 mm, the diameter of the bar is 25 mm, the length of the parallel part in the middle is 30 mm, and the diameter is 10 mm.

### 2.2. Experimental Study

**Figure 2:**
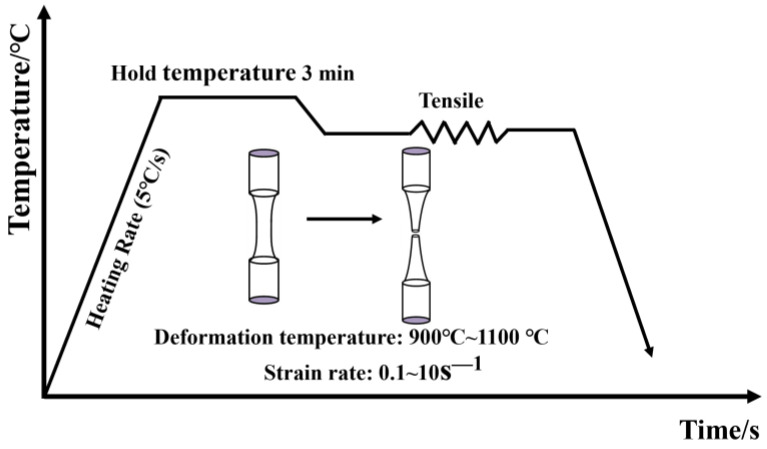
Uniaxial thermal tensile test flow.

The forged railway wagon bogie adapter was machined into the Figure 1 dimensions by means of an electric spark cutting machine and a CNC lathe. The high temperature tensile test was carried out with a Gleeble-1500D thermal simulator, Dynamic Systems Inc., Poestenkill, NY, USA. As shown in Figure 2, the sample was heated to 1200 °C at 5 °C/s and kept warm for 3 min so that it was fully austenitized. It was then cooled to 1100, 1000, 900 °C at a cooling rate of 5 °C/s, respectively, and isothermal stretching was carried out at a strain rate of 0.1, 1, and 10 s^−1^, respectively. After undergoing tension, the sample was rapidly water-cooled to preserve its microstructure.

## 3. Results and Discussion

### 3.1. Introduction to GTN Model

Gurson proposed a body cell model with voids in a finite large matrix and obtained the yield functions applicable to two shapes of voids, cylindrical voids in a cylindrical cell and spherical voids in a three-dimensional body cell, as shown in Equation (1).

(1) $$\Phi ={\left(\frac{\sigma_{eq}}{\sigma_{y}}\right)}^{2}+2fcosh\left(\frac{3\sigma_{m}}{2\sigma_{y}}\right)-\left(1+f^{2}\right)=0$$

Tvergaard and Needleman revised the Gurson model and proposed three modified parameters to describe the interaction between voids. The improved model is the GTN damage model that many researchers now use. The yield function of the GTN damage model is expressed as:

(2) $$\Phi ={\left(\frac{\sigma_{eq}}{\sigma_{y}}\right)}^{2}+2q_{1}f^{*}\cosh\left(\frac{3\sigma_{m}q_{2}}{2\sigma_{y}}\right)-\left(1+q_{3}{\left(f^{*}\right)}^{2}\right)=0$$

$f^{*}$ is a piecewise continuous function used to describe the through polymerization of microvoids in the necking process of a material, and $f^{*}$ = 0 when no damage occurs in the material; the functional relationship is as follows:

(3) $$f^{*}=\{\begin{array}{c} f\;\;\;\;\;\;\;\;\;\;\;\;\;\;\;\;\;\;\;\;\;\;\;\;\;f\leq f_{c}\\ f_{c}+k\left(f-f_{c}\right)\;\;f>f_{c} \end{array}$$

(4) $$k=\frac{f_{u}^{*}-f_{c}}{f_{f}-f_{c}}$$

In the formula, $f_{c}$ is the critical porosity, $f_{F}$ is the void fraction at the time of failure, and k is the void growth acceleration factor. The total damage consists of two parts: nucleation of newly formed voids, and the growth of voids that have been nucleated.

(5) $$f=f_{grow}+f_{nucl}$$

Assuming the voids surrounding the characteristic volume element are incompressible, their growth is contingent upon the plastic deformation of the matrix.

(6) $$f_{grow}=\frac{f_{n}}{S_{n}\sqrt{2\pi }}\exp\left(-\frac{1}{2}\left(\frac{\varepsilon_{m}-\varepsilon_{n}}{S_{n}}\right)\right)\varepsilon_{p}$$

where $f_{n}$ is the volume fraction of the nucleable void, $S_{n}$ is the standard deviation of void nucleation strain, and $\varepsilon_{n}$ is the mean nucleation strain.

### 3.2. Material Parameters in the GTN Model

According to the formula of the GTN damage model, there are nine undetermined values in the model: $f_{0}$, $f_{n}$, $f_{c}$, $f_{F}$, $q_{1},$ $q_{2},$ $q_{3}$, S_N_ and $\varepsilon_{N}$. Among them, $q_{1}$, $q_{2}$ and $q_{3}$ serve as correlation coefficients for material properties, are primarily influenced by material yield and hardening, and are calibrated based on the stress–strain curve. S_N_ and $\varepsilon_{N}$ reflect the inclusion and nucleation of voids in the material. The deviation between experiment and simulation can be used to calibrate GTN parameters. $f_{0}$, $f_{n}$, $f_{c}$ and $f_{F}$ are different stages of void evolution. Kiran and Khandelwal [27] proposed three methods to calibrate them, the third of which assumed that both the initial and nucleating voids were the cause of the damage. Therefore, the GTN model is corrected with the third hypothesis.

### 3.3. Establishment of Finite Element Model

**Figure 3:**
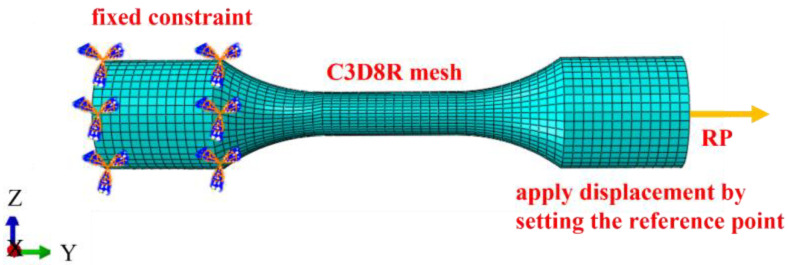
Tensile sample geometric model and mesh.

Using ABAQUS 2016 software, a numerical model for unidirectional tensile testing was created based on the actual dimensions of the sample, as illustrated in Figure 3, to simulate the entire unidirectional tensile test procedure. The simulation is performed in an Abaqus/Explicit dynamic module, where large displacements and deformations are allowed, while damage effects can be taken into account. The AISI 1035 Steel Elastic Model Emperor E and Patson values are 212,000 and 0.3, respectively, and the density is 7.85 kg/m^3^. The model selected porous metal plasticity to analyze the damage evolution, set the lower end as a fixed constraint, and applied displacement by setting a reference point above. Based on the GTN model, the high temperature tensile of AISI 1035 steel was simulated.

### 3.4. Calibration of $q_{1}$, $q_{2}$ and $q_{3}$

**Figure 4:**
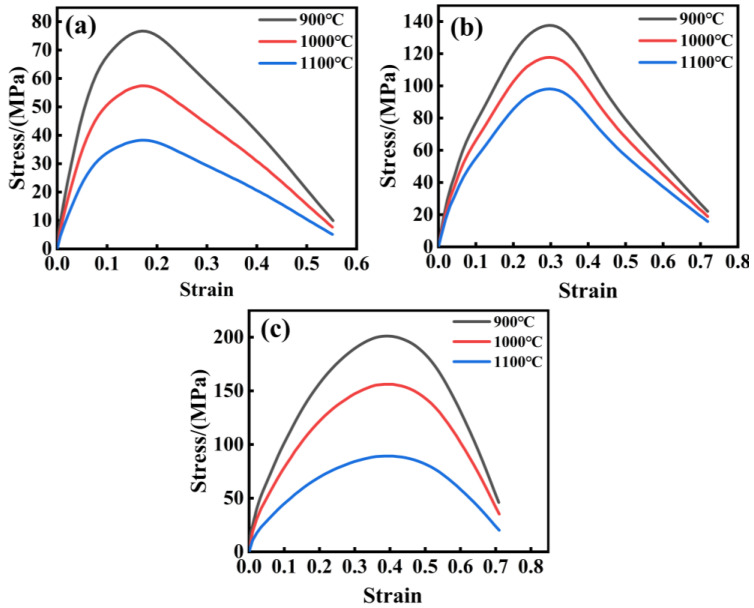
Stress–strain curve of AISI 1035 steel: (**a**) 0.1 s^−1^, (**b**) 1 s^−1^, and (**c**) 10 s^−1^.

Figure 4 depicts the actual stress–strain curve of AISI 1035 steel under varying temperatures and strain rates. During the elastic stage, the stress distribution within the material is fairly even, with no noticeable nucleation occurring. With the increase in stress, plastic deformation begins to appear in the material, and the local stress concentration area may trigger the nucleation of a hole. When the material enters the necking stage, the local stress concentration intensifies, and the holes grow rapidly and polymerize, eventually leading to the fracture of the material. At this moment, the stress value on the stress–strain curve gradually diminishes, while the strain value keeps on rising.

After the correction coefficients $q_{1}$, $q_{2}$ and $q_{3}$ were introduced, Faleskog [28] connected the correction coefficients with the ratio of the yield stress to elastic modulus,$\sigma_{y}$/*E*, and hardening index, as shown in Table 2 and Formula (7).

(7) $$\varepsilon =\frac{\sigma }{E}+\alpha {\left(\frac{\sigma }{E}\right)}^{n}$$

**Figure 5:**
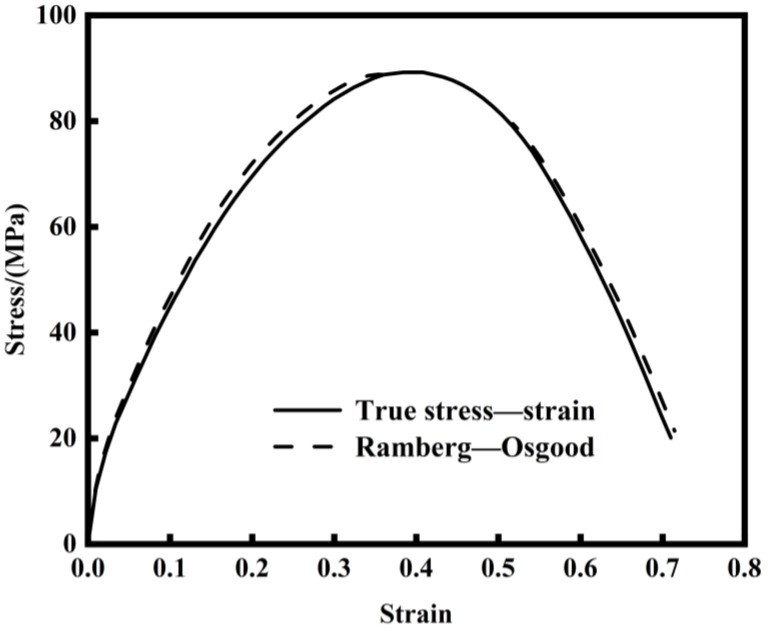
Fitting results of the Ramberg–Osgood hardening model.

The Ramberg–Osgood hardening model was employed to fit the tensile stress–strain curve, as shown in Figure 5. The resulting parameters α and n are listed in Table 2. Referring to Table 3 and Faleskog’s research, $q_{1}$ = 1.78, $q_{2}$ = 0.833, $q_{3}={q_{1}}^{2}=$3.17 is adopted for the GTN model of AISI 1035 steel.

### 3.5. Calibration of $\varepsilon_{n}$ and $S_{N}$

In the GTN model, $\varepsilon_{n}$ and $S_{N}$ determine the rate of void nucleation. Yin Y and Li [29]s’ research shows that $\varepsilon_{n}$ represents the plastic strain level at the beginning of the cracking macro damage. Therefore, the strain value at the departure of the finite element load–displacement curve from the experiment is used as the approximate value of $\varepsilon_{n}$.

**Figure 6:**
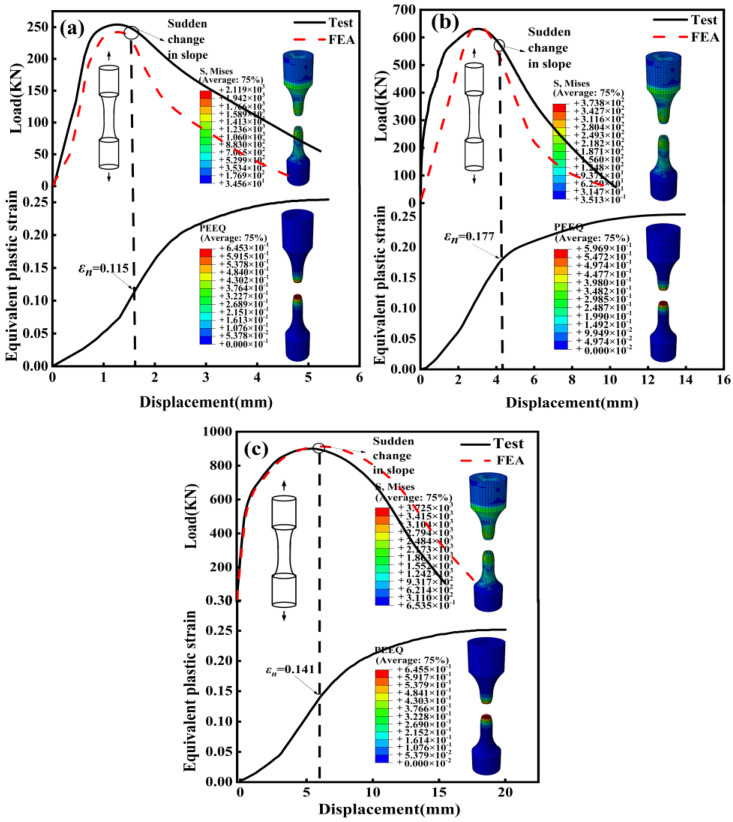
Calibration of void nucleation strain $\varepsilon_{N}$ of AISI 1035 steel: (**a**) 0.1 s^−1^; (**b**) 1s^−1^; (**c**) 10 s^−1^.

Peeq (plastic equivalent strain), which is an important index of the material in the plastic stage, is used to analyze the deformation behavior of the material. The void nucleation strain $\varepsilon_{n}$ of AISI 1035 steel is 0.115, 0.177 and 0.141, respectively, as shown in Figure 6a–c. Therefore, the average $\varepsilon_{n}$ = 0.144 of the GTN model of AISI 1035 steel is determined. The solution of $\;S_{N}$ is generally based on empirical methods, so according to previous experience, $\;S_{N}$ is 0.1 [30].

### 3.6. Calibration of $f_{0},\;f_{n},\;f_{c}$ and $f_{F}$

**Figure 7:**
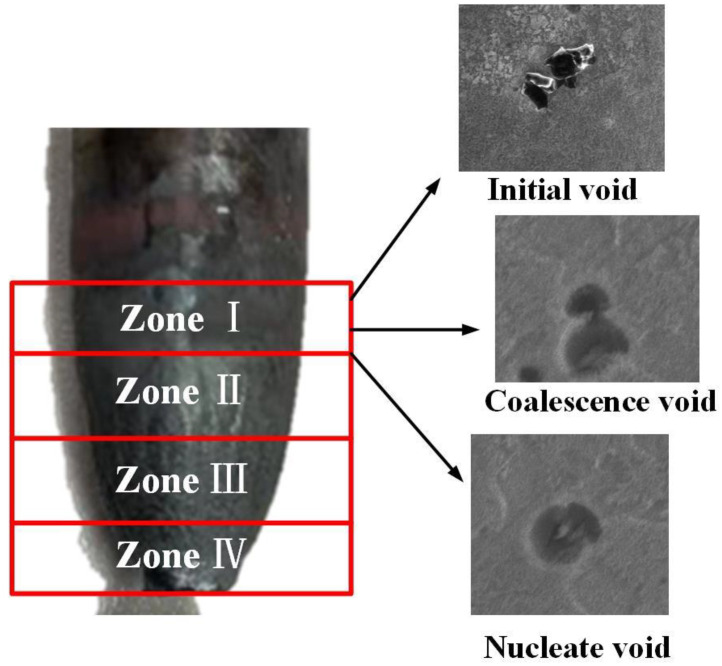
Initial voids in the volume fraction measurement area and the undeformed area of the neck of the stretched sample.

**Figure 8:**
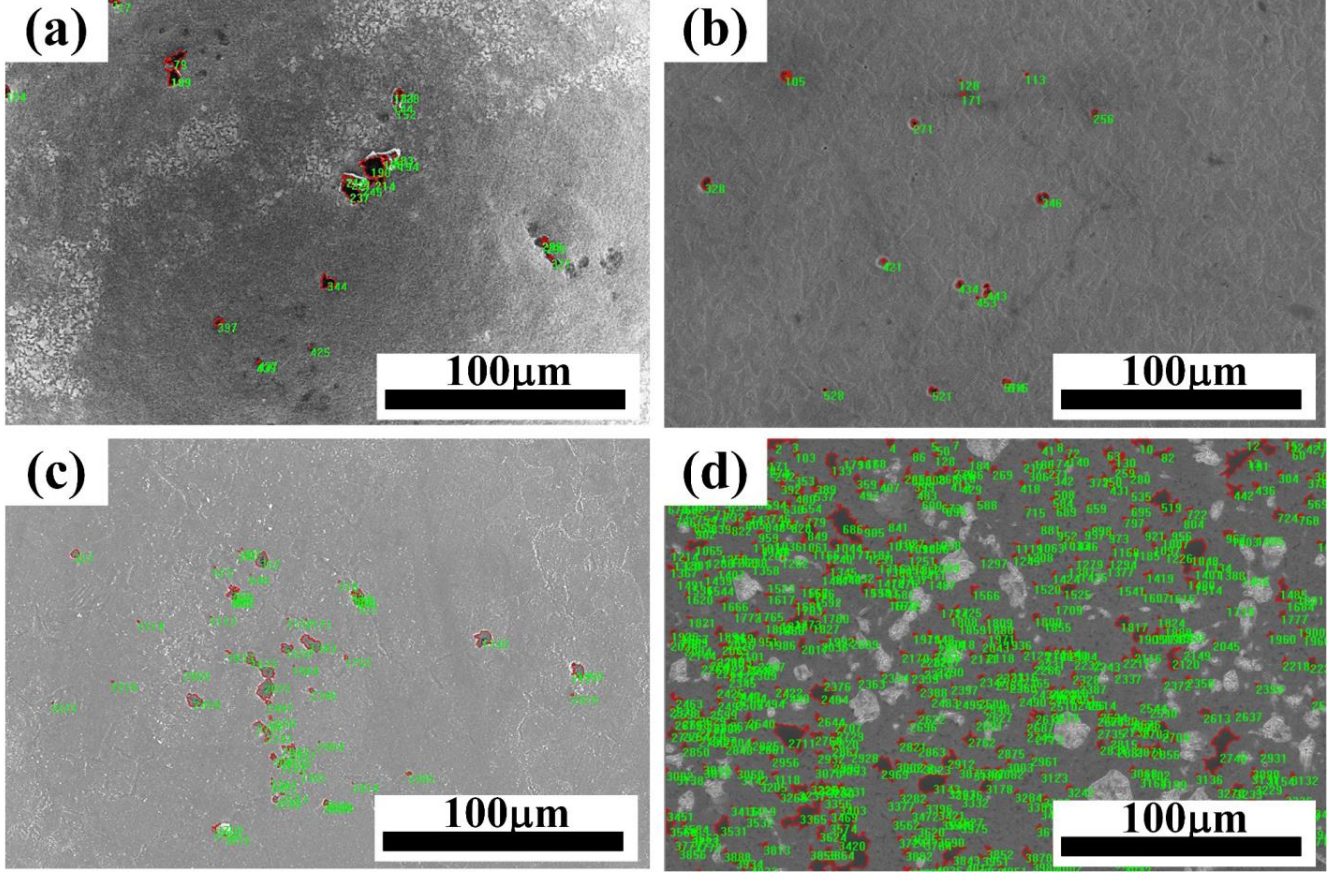
Digital image processing of SEM micrographs was used to calculate the porosity. (**a**) $f_{0}$; (**b**) $f_{n}$; (**c**) $f_{c}$; and (**d**) $f_{F}$.

The necking zone of the sample after tensile fracture is divided into four regions, which are the basis for calibration of the four parameters $f_{0},\;f_{n},\;f_{c}$ and $f_{F}$, as shown in Figure 7. Zone I is the undeformed zone, zone II is the uniform deformation zone, zone III is the adjacent fracture zone, and zone IV is the final failure zone. Figure 8a shows the material containing the initial void in the undeformed region of zone I. Image Pro Plus software was used to calculate the void proportion. The calculated $f_{0}$ = 0.0053.

**Figure 9:**
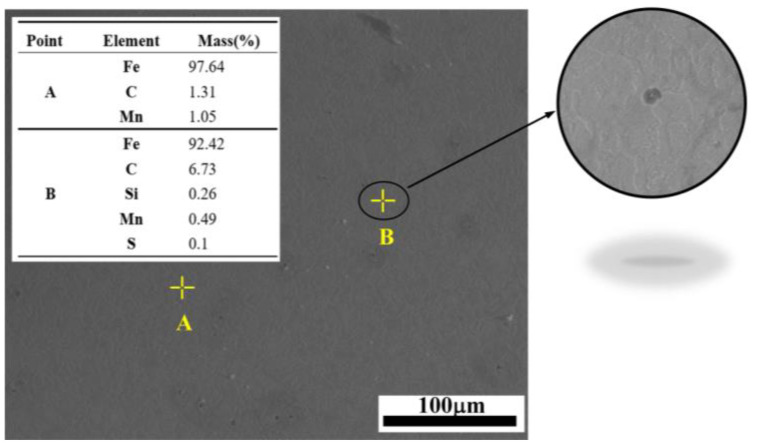
Matrix with partially grown void and nucleation void.

Second-phase particles within the matrix are essential for void nucleation. The calibration of $f_{n}$ depends on the ratio of these particles in zone II. The composition of the precipitated phase of AISI 1035 steel was analyzed by energy dispersive spectrometer. Figure 9 shows the analysis region and energy spectrum results.

The main elements of AISI 1035 steel are Fe, Si and Mn. According to the energy spectrum results of Figure 9, it is observed that second-phase particles like S and P are present, meeting the conditions necessary for void nucleation. Image Pro Plus software was used to identify the second-phase precipitates, as shown in Figure 8b, and the proportion of particles in the second phase was 0.027, that is $f_{n}$ = 0.027.

The voids coalesce before the final fracture [31]. The void proportion was calculated in region III as the value of critical void volume fraction $f_{c}$, as shown in Figure 8c, $f_{c}$ = 0.071. The calculation of fracture void volume fraction $f_{F}$ is based on zone IV, as shown in Figure 8d, and the void proportion was 0.085.

### 3.7. Damage Evolution Analysis

According to the mesoscopic damage theory, the continuous accumulation of damage can be considered as void nucleation, growth and aggregation. (1) The nucleation of microvoids: due to the fragmentation of two-phase particles or separation from the matrix material formed, mostly in the inclusion or two-phase particle interface. (2) Microvoid growth: As macroscopic plastic deformation increases, the number and volume of microvoids grow rapidly. (3) Coalescence of microvoids: The increased number and volume of microvoids lead to their coalescence, resulting in macroscopic cracks and material fracture [32].

**Figure 10:**
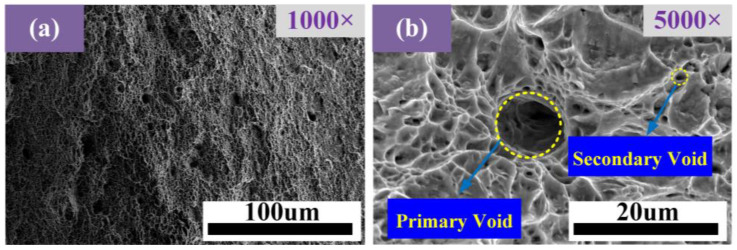
Fracture morphology of A1S1 1035 steel at 1100 °C, 10 s^−1^: (**a**) 1000×; and (**b**) 5000×.

To investigate the fracture characteristics of materials and understand how temperature and strain rate affect fracture, it is essential to analyze the fracture morphology of materials. As can be seen in Figure 10, there are dimples distributed at the fracture of the sample. The distinguishing feature of ductile fractures is the existence of dimples. It can be judged that the fracture mode of the materials is ductile fracture [33,34,35].

Figure 10a shows the fracture morphology of AISI 1035 steel at 1100 °C and 10 s^−1^ with a magnification of 1000. The microvoids in the material aggregate and connect with each other, and cracks appear when the proportion of voids increases to a certain extent. Figure 10b shows a 5000-fold fracture morphology. The primary dimple is large and the secondary dimple is small. Secondary voids will nucleate during deformation.

**Figure 11:**
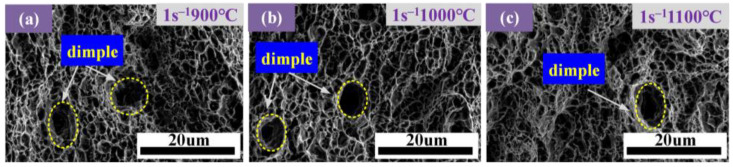
Fracture morphology of AISI 1035 steel tensile parts at different temperatures: (**a**) 900 °C; (**b**) 1000 °C; and (**c**) 1100 °C.

**Figure 12:**
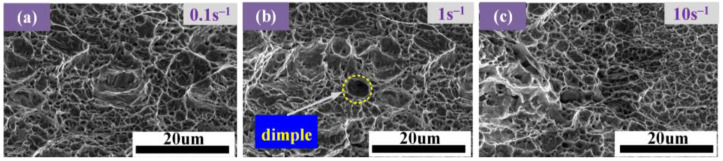
Fracture morphology of AISI 1035 steel tensile parts at different strain rates: (**a**) 0.1 s^−1^; (**b**) 1 s^−1^; and (**c**) 10 s^−1^.

Figure 11 and Figure 12 show the respective morphology of fracture samples under SEM. It can be seen from Figure 11 that the dimples are larger and deeper at 1100 °C, indicating that the second-phase particles are larger and that the material has strong plastic deformation ability. When the strain rate is 1 s^−1^, the dimples appear, indicating that the toughness at this strain rate is greater. However, this dimple is smaller and shallower than the above dimples, indicating that the temperature significantly influences the material’s toughness.

Based on the above observations, it can be found that the damage and fracture of ductile metal materials result from microvoids, and the internal impurities in the material nucleate, grow and polymerize under the influence of the external environment and load, resulting in damage and fracture. The SEM observations demonstrate the rationality of the GTN model in simulating the damage fracture of ductile metal materials.

### 3.8. Establishment of Response Surface Method

**Figure 13:**
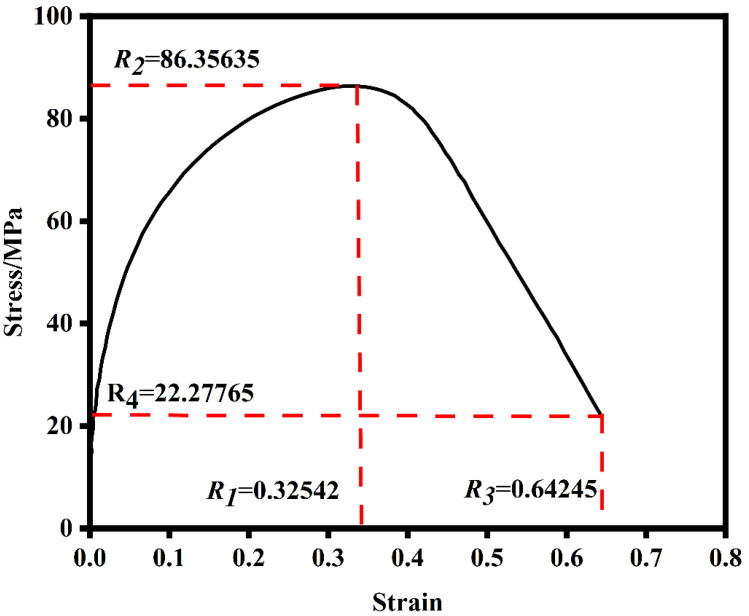
Response value of stress–strain curve.

Based on the GTN damage model, uniaxial tensile deformation experiments at high temperatures of 900, 1000, 1100 °C and strain rates of 0.1, 1, 10 s^−1^ were simulated by finite element simulation. When the response value was selected, the four points of the stress peak point and the transverse and longitudinal coordinates of the failure point of the macro-stress–strain curve were set as R1, R2, R3 and R4 [36], and the stress–strain data of 0.1 s 1000 °C were selected as an example, as shown in Figure 13.

Based on the combination of four factors and three levels of test parameters, 29 test groups were designed using the orthogonal test method. The data were obtained from the stress–strain curves obtained by finite element simulation. Table 4 displays the selected damage parameter grades. Following this experimental design and the use of Design expert 13 software, this experiment selected 1100 °C as an example. The experimental results are detailed in Table 5.

#### 3.8.1. Response Surface Model Fitting and Significance Analysis Test

The variance analysis of the regression model is shown in Table 6. In this analysis, the P-values for each response variable R1, R2, R3, and R4 are extremely close to zero, indicating that the model has very significant statistical significance. The response model formula is shown in Equations (8)–(11).

(8) $$\begin{array}{c} R1=0.480960-1.17636f_{0}+0.086295f_{n}+0.105956f_{c}-0.036462f_{F}\\ \;\;-11.60909f_{0}*f_{n}-8.28267f_{0}*f_{c}+13.35200f_{0}*f_{F}\\ \;\;-1.06010f_{n}*f_{c}+0.002045f_{n}*f_{F}-0.417333f_{c}*f_{F} \end{array}$$

(9) $$\begin{array}{c} R2=121.51502-173.17333f_{0}+71.63398f_{n}-7.27615f_{c}-8.47192f_{F}\\ \;\;+7096.16364f_{0}*f_{n}+34.44452f_{0}*f_{c}+48.8625f_{0}*f_{F}\\ \;\;-7.200865f_{n}*f_{c}-15.255156f_{n}*f_{F}-0.4767f_{c}*f_{F} \end{array}$$

(10) $$\begin{array}{c} R3=0.713056-10.63087f_{0}-0.379224f_{n}-0.188299f_{c}-0.060719f_{F}\\ \;\;+90.377f_{0}*f_{n}+11.61813f_{0}*f_{c}+0.683308f_{n}*f_{c}-1.36128f_{n}*f_{F}\\ \;\;-0.0744f_{c}*f_{F}-2.73168f_{0}*f_{F}+1256.44640{f_{0}}^{2}+5.11924{f_{n}}^{2}\\ \;\;+2.61542{f_{c}}^{2}+0.322938{f_{F}}^{2} \end{array}$$

(11) $$\begin{array}{c} R4=32.17556-461.27990f_{0}-7.15611f_{n}-4.85988f_{c}-11.20834f_{F}\\ \;\;+955.45455f_{0}*f_{n}-1977.77778f_{0}*f_{c}+3225.86667f_{0}*f_{F}\\ \;\;+263.58586f_{n}*f_{c}-7.24242f_{n}*f_{F}+45.17037f_{c}*f_{F} \end{array}$$

#### 3.8.2. Response Surface Analysis

**Figure 14:**
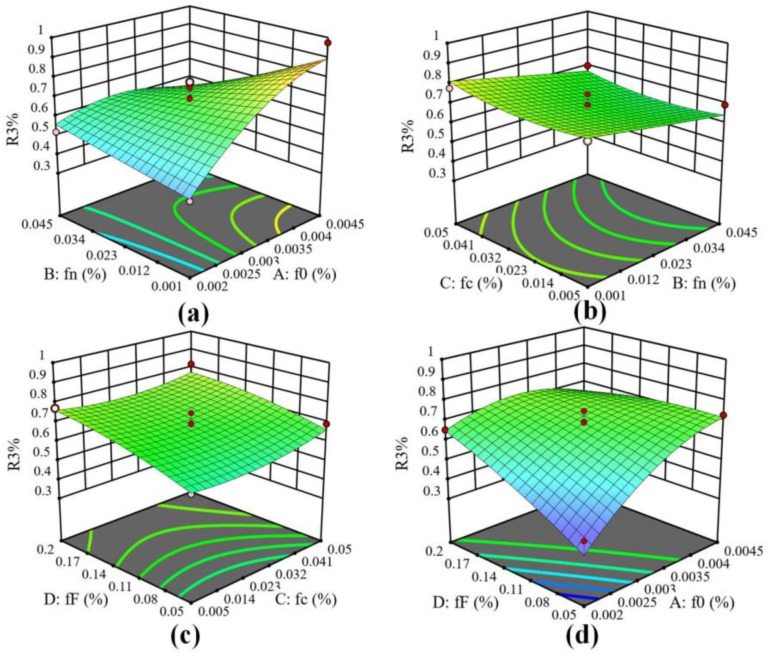
Response surface model of the effect of different parameter combinations on material strain at breaking point (R3). (**a**) Combination of $f_{0}$ and $f_{n}$, (**b**) Combination of $f_{n}$ and $f_{c}$, (**c**) Combination of $f_{c}$ and $f_{F}$, (**d**) Combination of $f_{F}$ and $f_{0}$.

The three-dimensional response surface of fracture strain affected by four significant factors $f_{0}$(A), $f_{n}$(B), $f_{c}$(C) and $f_{F}$(D) was drawn by Design-Expert software, as shown in Figure 14. The figure shows that with the decrease in $f_{0}$ and the increase in $f_{n}$, R3 generally tends to decrease, which may be due to the increase in the proportion of $f_{n}$ caused by partial void nucleation (Figure 14a). R3 decreases with the increase in $f_{n}$ and the decrease in $f_{c}$. The reason is that there are more nucleated voids and the voids coalesce, and that the time for the material to reach the necking state is shorter [37] (Figure 14b). With the decrease in $f_{c}$ and $f_{F}$, R3 decreases, indicating that the void size increases rapidly and that the fracture occurs soon after necking (Figure 14c). The effect of $f_{F}$ on R3 is small because of the short time between necking and fracture (Figure 14d).

The interaction of each damage parameter significantly affects the void volume fraction of materials. Table 7 displays the damage parameters obtained based on the predictions of the quadratic regression model.

### 3.9. Finite Element Simulation Analysis

**Figure 15:**
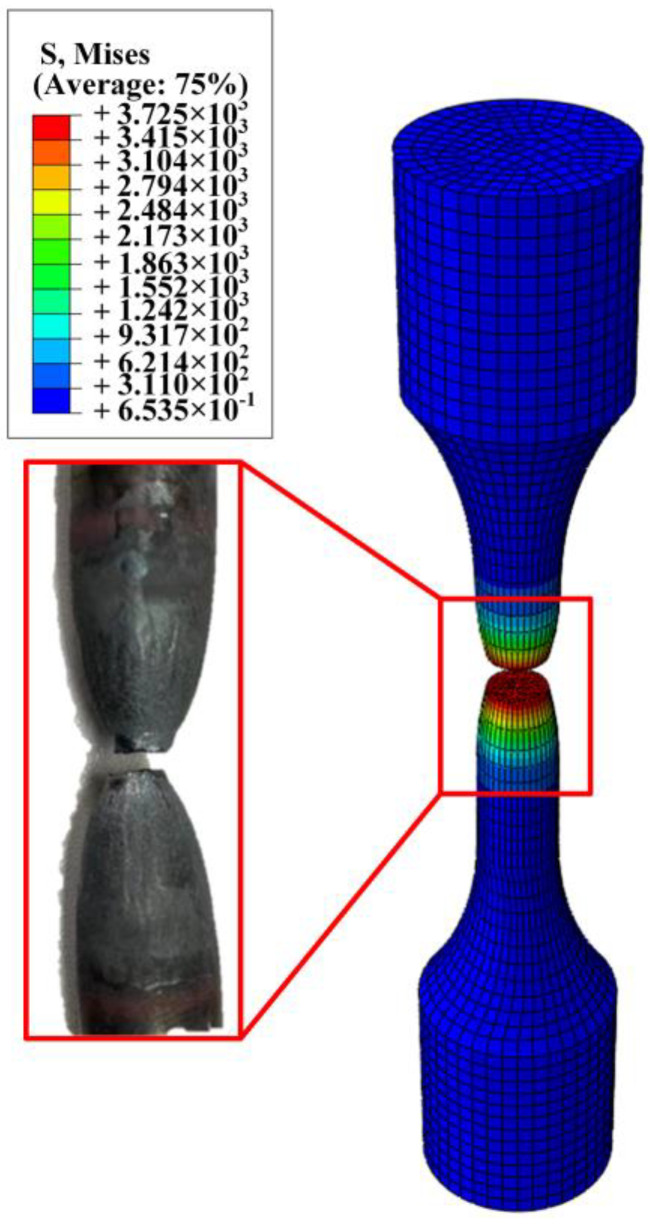
The 35 steel’s final fracture zone was obtained by experiment and finite element analysis.

**Figure 16:**
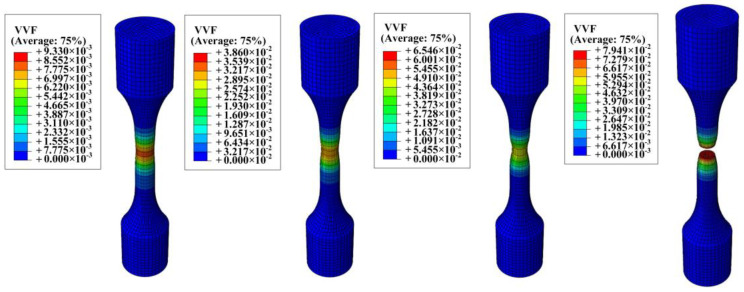
The 1100 °C thermal tensile simulation void volume fraction (VVF) distribution cloud image.

As depicted in Figure 15, the fracture location of the sample is compared with the results from finite element simulations, confirming the accuracy of the finite element analysis. Figure 16 shows VVF (Void Volume Fraction) cloud images in four stages. First of all, the porosity increases from 0.00077 to 0.0093, and the damage is concentrated in the central area. When the porosity increases to 0.0386, the sample shows an obvious necking phenomenon, and when the porosity reaches 0.0654, the sample reaches the near-fracture state. When the sample breaks, the porosity reaches 0.079. The porosity of the four stages corresponds to the porosity calibrated by the experimental method. The error is within 10%, which meets the requirements.

**Figure 17:**
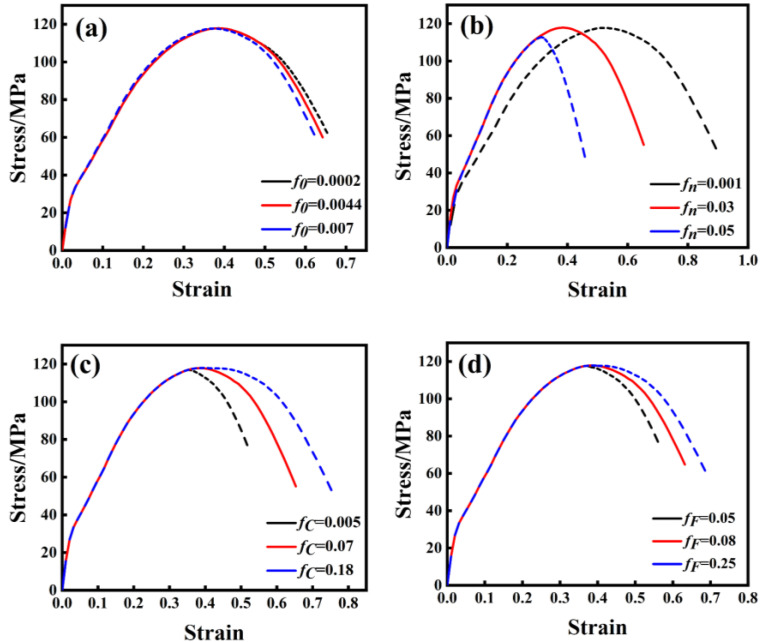
Impact of each damage parameter on the tensile curve. (**a**) *f*_0_, (**b**) $f_{n}$, (**c**) $f_{c}$, (**d**) $f_{F}$.

$f_{0}$ has little effect on tensile curve shape and fracture strain, as shown in Figure 17a. According to Figure 17b, it is evident that $f_{n}$ exerts a significant impact on the mechanical properties of the material, particularly affecting its fracture strain and tensile strength. Higher values of $f_{n}$ lead to increased nucleation of inclusions within the material, resulting in lower tensile strength and corresponding strain at failure, thus diminishing the material’s mechanical properties [38,39]. The primary effect of $f_{c}$ on the tensile curve is observed in Figure 17c: larger values of $f_{c}$ correspond to slower void polymerization times, resulting in higher tensile strength and corresponding strain at failure. This improvement reflects enhanced mechanical properties of the material [40]. The tensile curve’s fracture stress and strain are predominantly influenced by $f_{F}$ [41]. The smaller $f_{F}$ is, the more likely the material is to fracture, as shown in Figure 17d.

## 4. Conclusions

This paper mainly introduces the fracture mechanism of AISI 1035 steel and the micro-fracture model based on voids. The specific conclusions are as follows:

(1) The method of fitting the stress–strain curve of AISI 1035 steel with the Ramberg–Osgood hardening model is used to calibrate $q_{1}$ = 1.78, $q_{2}$ = 0.833, $q_{3}={q_{1}}^{2}$ = 3.17 in the GTN model. The inflection point of the curve slope obtained by the finite element method and experimental method is used to determine $\varepsilon_{N}$ = 0.144, and the deviation parameter $S_{N}$ is 0.1. The GTN model parameters which can characterize the damage of AISI 1035 steel were determined by combining the macroscopic mechanical properties with the microscopic morphology. By comparing the damage parameters obtained by the two methods, it is proved that the fracture parameters obtained can better simulate the fracture of materials, and the GTN fracture model exhibits strong applicability to the fracture of AISI 1035 steel. The error of the damage parameters obtained by the two methods is less than 10%, confirming the GTN damage model’s effectiveness in predicting the evolution of damage behavior in AISI 1035 steel.
(2) By observing the fracture morphology of AISI 1035 steel, it is found that the size and depth of the dimples of the sample at 1100 °C are larger and deeper, which reveals that AISI 1035 steel has good plastic deformation ability and shows typical ductile fracture characteristics.
(3) By studying the effects of different damage parameters on fractures, it is found that the effect of $f_{0}$ on fractures is relatively small, while the effects of $f_{n}$, $f_{c}$ and $f_{F}$ on fractures are larger. The larger $f_{n}$ is, the earlier the fracture point is; conversely, the larger $f_{c}$ and $f_{F}$ are, the later the fracture occurs.

Accurate GTN model parameters enable the prediction of the mechanical behavior of 1035 steel under various loading conditions, particularly during plastic deformation and fracture. By developing a precise damage model, researchers can optimize the forging process at the design stage, thereby enhancing both product performance and reliability. In summary, calibrating the GTN model parameters offers significant theoretical support and practical guidance for improving the performance of 1035 steel in forging and other applications.

## Figures and Tables

**Table 1:** Composition of AISI 1035 steel.

Element	C	Mn	Si	S	P
Wt.%	0.32	0.79	0.89	0.01	0.021

**Table 2:** Values of *q*_1_ and *q*_2_ suggested by Faleskog et al.

Hardening N(1/*n*)	*σ_y_*/*E* = 1 × 10^−3^		*σ_y_*/*E* = 2 × 10^−3^		*σ_y_*/*E* = 4 × 10^−3^	
	*q*_1_	*q*_2_	*q*_1_	*q*_2_	*q*_1_	*q*_2_
0.025	1.88	0.956	1.84	0.977	1.74	1.013
0.05	1.63	0.95	1.57	0.974	1.48	1.013
0.075	1.52	0.937	1.45	0.96	1.33	1.004
0.1	1.58	0.902	1.46	0.931	1.29	0.982
0.15	1.78	0.833	1.68	0.856	1.49	0.901
0.2	1.96	0.781	1.87	0.8	1.71	0.836

**Table 3:** Performance parameters of AISI 1035 steel.

Mechanical Properties	Test Results
Elastic modulus *E*	212,000 MPa
Yield strength *σ_y_*	341 MPa
Tensile strength *σ_u_*	587 MPa
Elongation *δ*	29.3%
*α*	0.21
*n*	7

**Table 4:** The selection level of high temperature damage parameters.

Level	*f*_0_	*f_n_*	*f_c_*	*f_F_*
1	0.002	0.001	0.005	0.05
2	0.00325	0.023	0.0275	0.125
3	0.0045	0.045	0.05	0.2

**Table 5:** The 1100 °C response surface design scheme and results.

No.	*f*_0_	*f_n_*	*f_c_*	*f_F_*	R_1_	R_2_	R_3_	R_4_
1	0.002	0.045	0.0275	0.125	0.479965	123.024	0.685369	30.6241
2	0.002	0.023	0.05	0.125	0.479404	121.281	0.68642	30.6601
3	0.0045	0.023	0.05	0.125	0.478994	121.014	0.686072	30.3251
4	0.002	0.001	0.0275	0.125	0.478415	119.872	0.690461	30.5728
5	0.00325	0.045	0.05	0.125	0.479066	122.728	0.68706	30.6726
6	0.002	0.023	0.0275	0.2	0.47769	120.898	0.685855	30.3273
7	0.0045	0.045	0.0275	0.125	0.479289	122.757	0.689301	30.4502
8	0.00325	0.001	0.05	0.125	0.479304	119.572	0.686447	30.3121
9	0.002	0.023	0.0275	0.05	0.480702	122.99	0.688677	30.8763
10	0.00325	0.045	0.0275	0.05	0.479888	123.445	0.691153	30.5225
11	0.00325	0.045	0.0275	0.2	0.479372	122.333	0.68332	30.5486
12	0.00325	0.023	0.005	0.05	0.478582	122.024	0.687645	30.5271
13	0.0045	0.001	0.0275	0.125	0.479016	119.604	0.684457	30.2938
14	0.00325	0.023	0.0275	0.125	0.479599	121.391	0.682919	30.4796
15	0.00325	0.001	0.0275	0.05	0.479021	120.291	0.686677	30.3994
16	0.00325	0.023	0.0275	0.125	0.479204	121.326	0.682955	30.4869
17	0.0045	0.023	0.005	0.125	0.479447	121.34	0.685402	30.4182
18	0.0045	0.023	0.0275	0.05	0.478164	121.737	0.688163	30.0432
19	0.002	0.023	0.005	0.125	0.478925	121.614	0.687057	30.5307
20	0.00325	0.023	0.05	0.05	0.480348	121.703	0.687878	30.3942
21	0.00325	0.023	0.0275	0.125	0.479246	120.99	0.682962	30.4528
22	0.00325	0.023	0.0275	0.125	0.479944	121.302	0.683	30.5087
23	0.00325	0.001	0.0275	0.2	0.478492	119.185	0.687829	30.4733
24	0.00325	0.001	0.005	0.125	0.478289	119.903	0.687167	30.5539
25	0.00325	0.045	0.005	0.125	0.48015	123.052	0.686427	30.3925
26	0.00325	0.023	0.0275	0.125	0.479035	121.356	0.682964	30.4445
27	0.00325	0.023	0.05	0.2	0.477947	120.596	0.684295	30.5989
28	0.0045	0.023	0.0275	0.2	0.480159	120.629	0.684317	30.7039
29	0.00325	0.023	0.005	0.2	0.478998	120.924	0.684568	30.4269

**Table 6:** Analysis of variance at different temperatures.

Temperature	R1	R2	R3	R4	R1	R2	R3	R4
	R^2^ (%)				*p*-Value			
900 °C	98.36	97.49	98.74	96.55	<0.0001	<0.0001	<0.0001	<0.0001
1000 °C	96.24	96.48	96.48	98.14	<0.0001	<0.0001	<0.0001	<0.0001
1100 °C	96.88	98.66	97.41	99.17	<0.0001	<0.0001	<0.0001	<0.0001
	Lack of Fit *p*-Value							
900 °C	0.363	0.625	0.065	0.079				
1000 °C	0.064	0.241	0.215	0.625				
1100 °C	0.099	0.957	0.278	0.815				

**Table 7:** Values of damage parameters under different conditions.

Rate, s^−1^ Temp, °C	0.1			1			10		
	900	1000	1100	900	1000	1100	900	1000	1100
$f_{0}$	0.003	0.0035	0.0042	0.003	0.0032	0.00425	0.0033	0.00365	0.0045
$f_{n}$	0.013	0.0524	0.0381	0.0082	0.0496	0.03496	0.0023	0.04633	0.0312
$f_{c}$	0.028	0.0152	0.0321	0.0424	0.0249	0.04588	0.0432	0.03156	0.0752
$f_{F}$	0.121	0.1852	0.0994	0.0983	0.1813	0.08195	0.0823	0.17915	0.0782

## Data Availability

No data or materials are available for this research. No codes are available for this research.
